# Strain-dependent induction of primary bile acid 7-dehydroxylation by cholic acid

**DOI:** 10.1186/s12866-024-03433-y

**Published:** 2024-08-01

**Authors:** Eduard Vico-Oton, Colin Volet, Nicolas Jacquemin, Yuan Dong, Siegfried Hapfelmeier, Karin Lederballe Meibom, Rizlan Bernier-Latmani

**Affiliations:** 1 Environmental Microbiology Laboratory, School of Architecture, Civil and Environmental Engineering, École Polytechnique Fédérale de Lausanne, Lausanne, Switzerland; 2 Institute for Infectious Diseases, University of Bern, Bern, Switzerland; 3 EPFL ENAC IIE EML CH A1 375 (Bâtiment CH), Station 6, CH-1015 Lausanne, Switzerland

**Keywords:** 7-dehydroxylation, *Clostridium scindens*, *Extibacter muris*, Gut microbiome, Deoxycholic acid (DCA), Lithocholic acid (LCA), Muricholic acid (MCA), Ursodeoxycholic acid, Cholic acid (CA), *Bai* gene expression, Conjugated bile acids

## Abstract

**Background:**

Bile acids (BAs) are steroid-derived molecules with important roles in digestion, the maintenance of host metabolism, and immunomodulation. Primary BAs are synthesized by the host, while secondary BAs are produced by the gut microbiome through transformation of the former. The regulation of microbial production of secondary BAs is not well understood, particularly the production of 7-dehydroxylated BAs, which are the most potent agonists for host BA receptors. The 7-dehydroxylation of cholic acid (CA) is well established and is linked to the expression of a bile acid-inducible (*bai*) operon responsible for this process. However, little to no 7-dehydroxylation has been reported for other host-derived BAs (e.g., chenodeoxycholic acid, CDCA or ursodeoxycholic acid, UDCA).

**Results:**

Here, we demonstrate that the 7-dehydroxylation of CDCA and UDCA by the human isolate *Clostridium scindens* is induced when CA is present, suggesting that CA-dependent transcriptional regulation is required for substantial 7-dehydroxylation of these primary BAs. This is supported by the finding that UDCA alone does not promote expression of *bai* genes. CDCA upregulates expression of the *bai* genes but the expression is greater when CA is present. In contrast, the murine isolate *Extibacter muris* exhibits a distinct response; CA did not induce significant 7-dehydroxylation of primary BAs, whereas BA 7-dehydroxylation was promoted upon addition of germ-free mouse cecal content *in vitro*. However, *E. muris* was found to 7-dehydroxylate *in vivo*.

**Conclusions:**

The distinct expression responses amongst strains indicate that *bai* genes are regulated differently. CA promoted *bai* operon gene expression and the 7-dehydroxylating activity in *C. scindens* strains. Conversely, the *in vitro* activity of *E. muris* was promoted only after the addition of cecal content and the isolate did not alter *bai* gene expression in response to CA. The accessory gene *baiJ* was only upregulated in the *C. scindens* ATCC 35704 strain, implying mechanistic differences amongst isolates. Interestingly, the human-derived *C. scindens* strains were also capable of 7-dehydroxylating murine bile acids (muricholic acids) to a limited extent. This study shows novel 7-dehydroxylation activity *in vitro* resulting from the presence of CA and suggests distinct *bai* gene expression across bacterial species.

**Supplementary Information:**

The online version contains supplementary material available at 10.1186/s12866-024-03433-y.

## Introduction

**Fig. 1:**
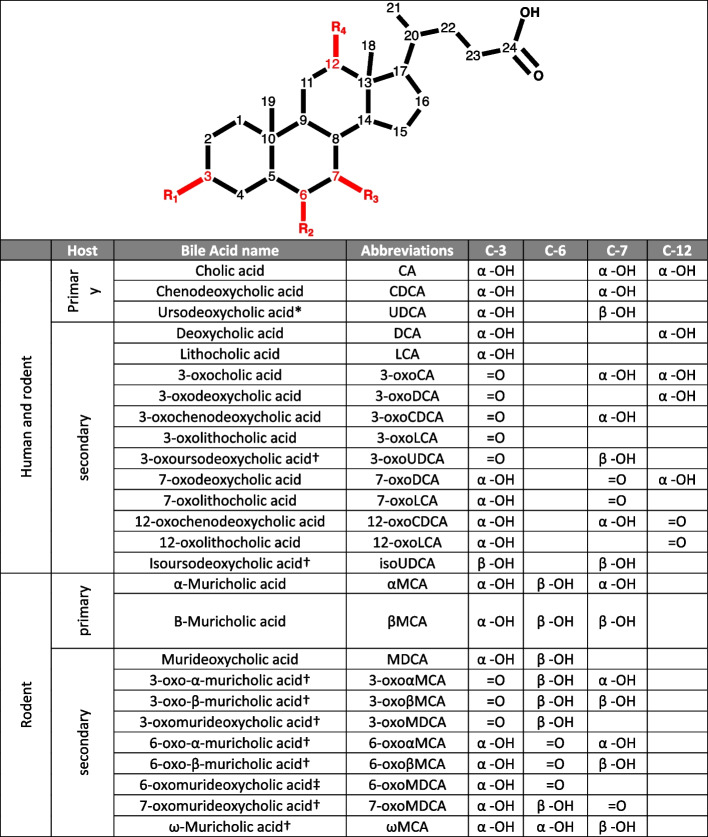
List of deconjugated human and rodent bile acids (BAs) discussed here. The characteristic that distinguishes BAs is the presence of a hydroxyl group at the C-3, C-6, C-7 and/or C-12 position. The hydroxyl groups can be in α- or β- conformation, oxidized into a ketone group, or fully removed (dehydroxylated BA). CA and CDCA are primary BAs of both humans and rodents whereas MCAs are exclusively produced by rodents. (*) UDCA is a primary BA in rodents while it is a secondary BA in humans where the gut microbes epimerize it from CDCA. (†) Bile acids that might have been detected in this study but for which no standards were available. (‡) Also known as 6-oxolithocholic acid (6-oxoLCA). See Supplementary Table 3 for full chemical names of the BAs

Primary bile acids (BAs) are metabolites synthesized from cholesterol by hepatocytes while secondary BAs are produced by the gut microbiome through the transformation of primary BAs (Fig. 1). In the liver, the BA are conjugated to glycine or taurine. The three main microbial BA transformations are deconjugation (loss of the amino acid group), oxidation (of one or several of the hydroxyl groups), and 7α-dehydroxylation (7-DH-ion), the loss of a hydroxyl group at the C7 position [1]. These microbial transformations increase the diversity of the BA pool (Fig. 1) and enhance BA affinity to host receptors. In particular, 7-DH-ion turns primary BAs such as cholic acid (CA) and chenodeoxycholic acid (CDCA) into the 7-dehydroxylated (7-DH-ed) BAs deoxycholic acid (DCA) and lithocholic acid (LCA), respectively [2].

BAs act as detergents to solubilize dietary fats, but also have important metabolic and immunomodulatory roles through activation of their target receptors [3]. The two best-studied BA receptors are the Farnesoid X Receptor (FXR), a nuclear receptor, and the G Protein-Coupled Bile Acid Receptor, GPBAR1, also known as Takeda G-Protein Receptor 5 (TGR5), which is a membrane receptor. FXR is activated through the binding of BA agonists, particularly the 7-DH-ed LCA but also DCA [4]. FXR activation results in the inhibition of primary BA synthesis through repression of the cholesterol 7α-hydroxylase CYP7A1. Regulation of BA production limits BA concentration and therefore, toxicity. On the other hand, BA dysregulation can cause health issues such as cholestasis, irritable bowel syndrome, gallstone disease, or even the induction of colorectal cancer [5–7]. Besides BA homeostasis, FXR also has focal roles in glucose and lipid homeostasis [8]. Similarly, TGR5 is a multifunctional regulator involved in glucose homeostasis, energy expenditure, and the modulation of the inflammatory response [9, 10]. LCA, DCA and their tauro-conjugated forms TLCA and TDCA, are among the strongest agonists of TGR5 [11, 12], highlighting the importance of microbial transformation, particularly 7-DH-ion, in TGR5 activation. Moreover, DCA and LCA may have protective properties against *Clostridium difficile* infection [13, 14].

The study of BAs has traditionally been based on mouse models [15]. Besides CA and CDCA, mice (and other rodents) also generate muricholic acids (MCAs) such as α-MCA and β-MCA (Fig. 1) and re-hydroxylate DCA and LCA in the liver [16]. Additionally, the mouse liver produces primary ursodeoxycholic acid (UDCA) [16] although there is evidence that the gut microbiome is responsible for a significant fraction of UDCA in the gut [14]. On the other hand, UDCA is exclusively a secondary BA in humans [7]. Because 7-DH-ion plays a major role in host homeostasis, significant effort has been expended to study 7-dehydroxylating (7-DH-ing) bacteria.

Nonetheless, experimental evidence of 7-DH-ion is limited to a few species of the *Clostridiales* order. One of the best characterized is the human isolate *Clostridium scindens* ATCC 35704, the type strain of *C. scindens* [17]. The ability of *C. scindens* ATCC 35704 to 7-dehydroxylate both *in vivo* and *in vitro* is well established [18, 19]. The *Extibacter muris* DSM 28560 (JM40) strain was recently isolated from mice and identified as a 7-DH-ing organism [20, 21]: it has been shown to 7-dehydroxylate *in vivo*, transforming the primary BAs CA, CDCA, αMCA, βMCA and UDCA into their respective secondary BAs DCA (from CA), LCA (from CDCA and UDCA) and MDCA (from αMCA and βMCA). Previous research had focused on *E. muris* strain DSM 28560 [22] and in this study, we demonstrate that *E. muris* strain DSM 28561 (SJ24) also has the ability to 7-dehydroxylate (7-DH-ate) *in vivo*.

**Fig. 2:**
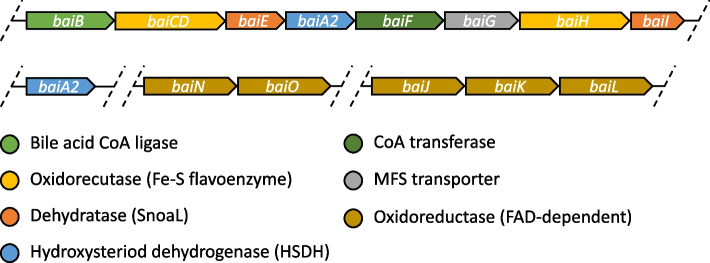
*bai* gene synteny. All the known *bai* genes are shown, with the main operon (*baiB-I*) and accessory genes shown below. However, not every 7-DH-ing bacteria has the full accessory gene set. Moreover, a novel *bai* gene synteny has been recently shown to also 7-dehydroxylate [31, 32]. The dehydratases contain a SnoaL-like domain

The biochemical machinery for 7-DH-ion is encoded in the *bai* (bile acid inducible) eight-gene operon (*baiBCDEA2FGHI*) [23, 24]). The CA 7-DH-ion pathway consists of multiple steps requiring six enzymes (BaiB, BaiCD, BaiE, BaiA2, BaiF, BaiH) and a bile acid transporter (BaiG), all encoded in the *bai* operon [24]. However, at least for CDCA, certain enzymes can be replaced by Bai proteins encoded outside the operon [19]. The *C. scindens* ATCC 35704 strain harbours the accessory gene *baiJ* (HDCHBGLK_03451) [19, 25] whereas the *E. muris* DSM 28650 genome includes a *baiJKL* pseudogene cluster [22] (Fig. 2). BaiJ has recently been shown to play a critical role in 7-DH-ion in *C. scindens* ATCC 35704 [19] but the other accessory genes *baiK* and *baiL* still do not have a clear assigned role and are not necessarily present in all BA 7-DH-ing strains [25]. Most of the published work on the 7-DH-ion pathway has been performed with another *C. scindens* strain, VPI 12708 [24, 26], that harbours BaiJ as well (Fig. 2), albeit with limited homology (47% identity, 62% similarity) to BaiJ from strain ATCC 35704. However, 7-DH-ing bacteria exhibit varying efficiency in transforming CA *in vitro*. The *C. scindens* ATCC 35704 and VPI 12708 strains show rapid transformation to DCA while *E. muris* DSM 28560 has more limited activity [18, 21, 27]. Other known 7-DH-ing strains such as *Clostridium hylemonae* and *Peptacetobacter hiranonis* have been reported as harboring weak and strong activity, respectively [28] and a new strain of *P. hiranonis* recently isolated from dog faeces displayed *in vitro* 7-DH-ion at around 30% conversion of CA to DCA [29]. Notably, *in vitro* 7-DH-ion of other primary BAs has been reported to be minor (CDCA) or non-existent (MCAs and UDCA) [18, 22, 30].

The limited *in vitro* 7-DH-ion of primary BAs other than CA (i.e., CDCA and MCAs) is striking considering that secondary 7-DH-ed forms of these BAs are routinely detected at significant concentrations in the host [33, 34]. Most studies tackling *in vitro* primary bile acid 7-DH-ion consider each BA in isolation. In addition, significant upregulation of the *bai* operon in response to CA is well established [35, 36] but there is no information about the potential response of this operon to other primary BAs (i.e., CDCA and MCAs) and whether upregulation of the *bai* operon by CA also results in the transformation of the latter. We hypothesize that CA-dependent upregulation of the *bai* operon promotes 7-DH-ing activity of other BAs when they occur together with CA. Moreover, we posit that primary BAs other than CA cannot induce their own transformation.

Here, the bile acid profile and the expression of *bai* genes were measured *in vitro* for three strains in the presence of CA, CDCA, αMCA, βMCA and UDCA, with and without amendment of ^13^C-CA to test whether the upregulation of *bai* genes was exclusive to CA and whether CA-driven expression was sufficient to promote the 7-DH-ion of other BAs. The experiments were performed with three strains, the human isolates *C. scindens* ATCC 35704 and VPI 12708 and the murine isolate *E. muris* DSM 28561 (SJ24). The results show that the response to CA was strain-dependent. It was highly effective for *C. scindens* strains and sufficient to promote the transformation of other primary BAs*.* For *E. muris*, none of the BAs tested promoted 7-DH-ion, nevertheless, a positive effect was observed when the bacterium was co-cultured with a small amount of cecal content from germ-free mice, suggesting that signaling from the host may be responsible for the induction of 7-DH-ion in *E. muris* SJ24.

This work highlights the importance of the presence CA for the 7-DH-ion of other BAs. Moreover, results from *E. muris* SJ24 point at host-related differences whereby BAs may not be the key inducers for BA 7-DH-ion in the murine gut.

## Results

### *In vitro* bile acid transformation and impact of ^13^C-CA

Two human isolates *C. scindens* ATCC 35704 and *C. scindens* VPI 12708 and one murine isolate *E. muris* DSM 28561 (SJ24) were tested for their ability to 7-DH-ate human and mouse primary BAs *in vitro*. The human isolates were chosen due to their role as representative species and the murine isolate was selected due to *E. muris* SJ24 being the only 7-DH-ing murine strain isolated that grows rapidly (growth within 24 hours).

CA is known to induce the expression of *bai* genes (hence the name bile acid *inducible* genes) but it is unknown whether other BAs also promote gene expression. Thus, the goal of these experiments was to confirm CA 7-DH-ion and to investigate whether the 7-DH-ion of CDCA, UDCA and MCA (α and β) can occur in the absence of upregulation by CA. Moreover, parallel experiments were performed by amending the cultures with ^13^C-CA to test whether the 7-DH-ion of CDCA, αMCA, βMCA and UDCA could be induced by CA (Supplementary Table 1).

**Fig. 3:**
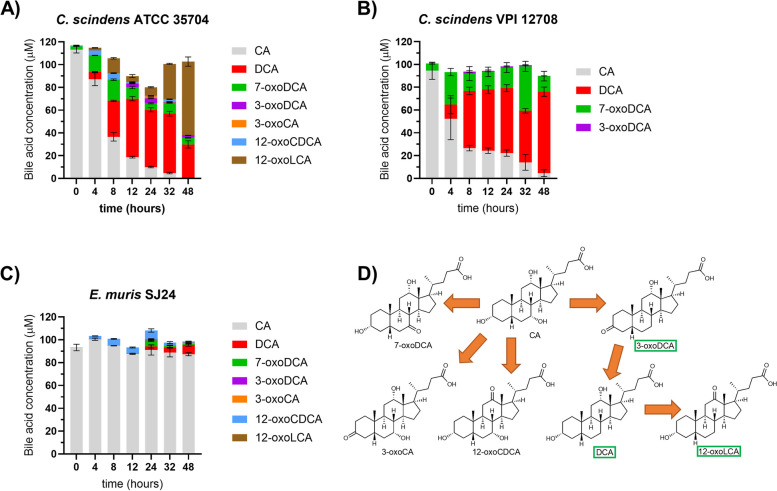
*In vitro* transformation of cholic acid. The 7-dehydroxylation of CA was tested in (**A**) *Clostridium scindens* ATCC 35704, (**B**) *C. scindens* VPI 12708 and (**C**) *Extibacter muris* DSM 28561 (SJ24) over time. (**D**) Possible cholic acid (CA) transformations, not necessarily all observed here. 7-dehydroxylated bile acids are marked by green boxes. All strains were grown anaerobically in BHIS-S containing 100 µM CA. Bile acids were extracted from the suspended biomass. Error bars represent the standard deviation of the mean of biological triplicates

As expected, all three strains 7-DH-ed CA but to varying extents (Fig. 3). *C. scindens* ATCC 35704 and *C. scindens* VPI 12708 showed strong 7-DH-ing activity with 97% and 80% CA conversion to 7-DH-ed BAs after 48 hours, respectively. *E. muris* SJ24, on the other hand, only converted 9% of the CA provided into 7-DH-ed forms (Fig. 3). *C. scindens* ATCC 35704 produced up to 52.26 µM DCA after 32 hours with some of the DCA subsequently oxidized to 12-oxolithocholic acid (12-oxoLCA) (Fig. 3A). In contrast, *C. scindens* VPI 12708 produced the highest amount of DCA after 48 hours (71.42 µM), with little to no oxidized DCA forms (0.19 µM of 3-oxoDCA at 48 hours) (Fig. 3B). The lack of 12-oxo forms from the *C. scindens* VPI 12708 strain was expected since the 12α-hydroxysteroid dehydrogenase (12α-HSDH) required for this process was not detected by PCR in this strain (the full genome is currently unavailable) (data not shown). Finally, *E. muris* SJ24 only produced 8.1 µM of DCA after 48 hours with very low amounts of oxidized forms of DCA (0.21 µM of 12-oxoLCA) (Fig. 3C). It is important to highlight that *E. muris* does not possess a 3α-HSDH encoded by *baiA2* which was recently identified as an important component of the CA 7-DH-ion pathway [24]. An alternative 3α-HSDH (BaiA1/3) has lower affinity to CA than BaiA2 [37] is likely present as *baiA1/3* was found outside the *bai* operon [38]. Finally, all three strains also showed a modicum of 7-oxidation activity (Fig. 3), resulting in the formation of 7-oxoDCA. This is an independent process from 7-DH-ion and the product cannot be 7-DH-ed.

**Fig. 4:**
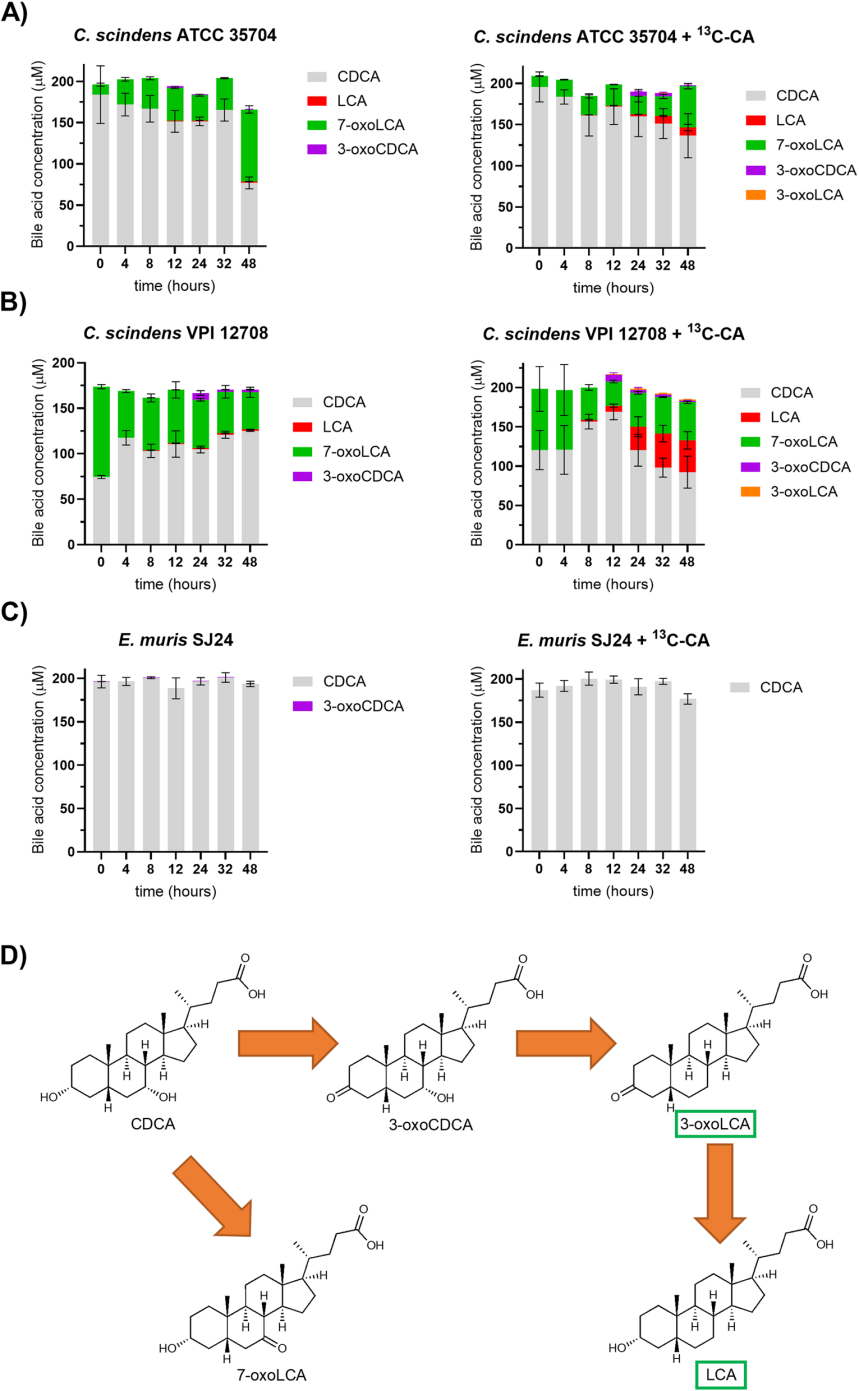
*In vitro* transformation of CDCA. The transformation of CDCA into secondary bile acids was tested with and without 100 µM of ^13^C-CA. 200 µM of CDCA were used based on previous experiments [18]. (**A**) *Clostridium scindens* ATCC 35704, (**B**) *C. scindens* VPI 12708 and (**C**) *E. muris* DSM 28561 (SJ24). (**D**) Possible chenodeoxycholic acid (CDCA) transformations, not necessarily all observed here (the CDCA 7-DH-ion pathway was recently described in Meibom et al., 2024). 7-dehydroxylated bile acids are marked by green boxes. Bile acids were extracted from the suspended biomass. Error bars represent the standard deviation of the mean of biological triplicates

CDCA 7α-dehydroxylation is very limited for all three strains. Indeed, *C. scindens* ATCC 35704 only produced 1.59 µM LCA and *C. scindens* VPI 12708 only 1.55 µM LCA (Fig. 4A and B), whereas for *E. muris*, no LCA was detected. The latter is in line with previous reports [22]. The amendment of ^13^C-CA significantly increased the transformation of CDCA for both *C. scindens* strains (*p-*value < 0.001 two-way ANOVA) but had no impact on *E. muris* SJ24 (Fig. 4C). Indeed, the LCA yield increased to 9.77 µM for strain ATCC 35704 and to 40.4 µM for strain VPI 12708 (Fig. 4A and B). No change was observed for *E. muris* SJ24.

**Fig. 5:**
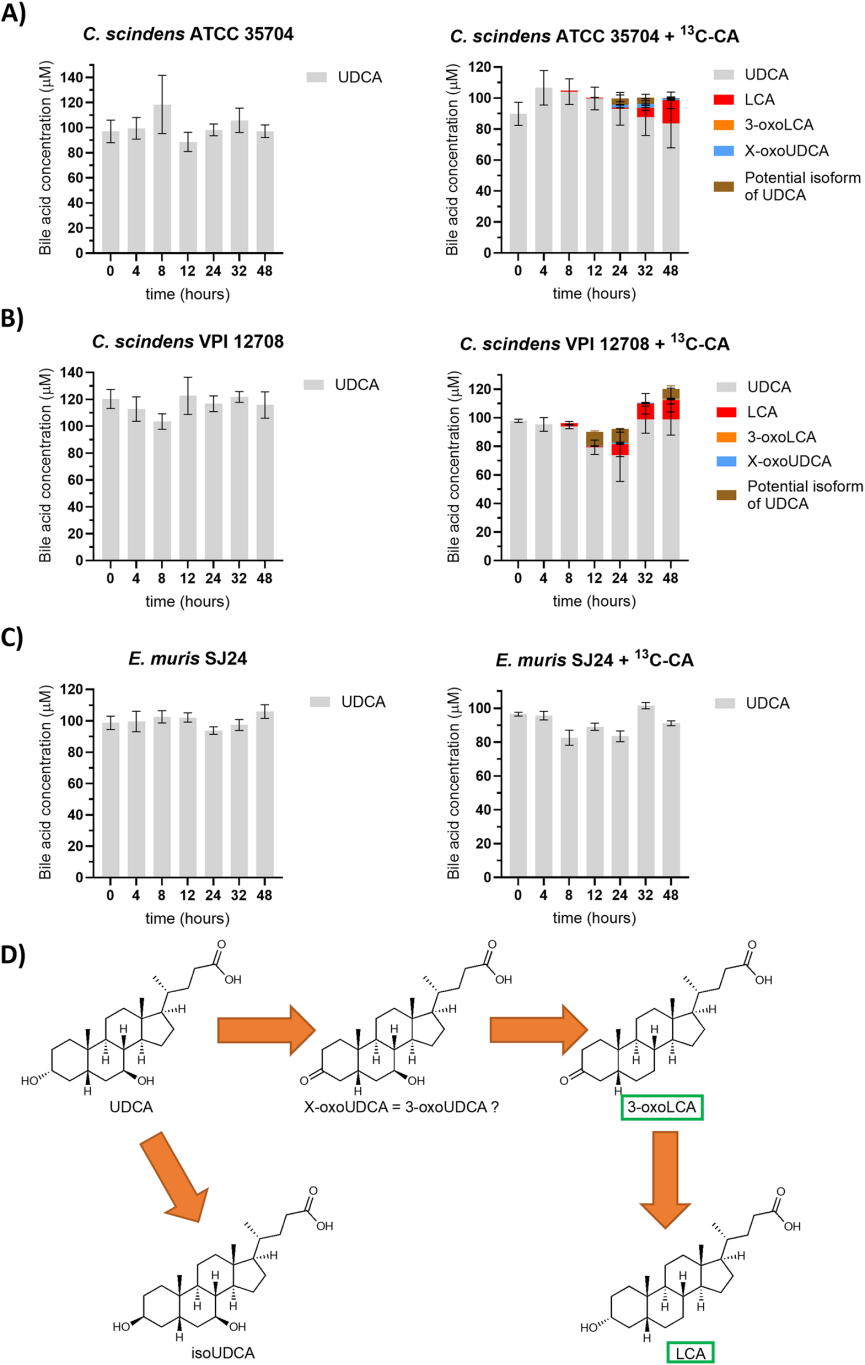
*In vitro* transformation of UDCA. The transformation of 100 µM of UDCA into secondary bile acids was tested with and without 100 µM of ^13^C-CA*.* **A** *Clostridium scindens* ATCC 35704, (**B**) *C. scindens* VPI 12708 and (**C**) *Extibacter muris* DSM 28561 (SJ24) were grown anaerobically in BHIS-S. Bile acids were extracted from suspended biomass. (**D**) Possible ursodeoxycholic acid (UDCA) transformations, not necessarily all observed here. 7-dehydroxylated bile acids are marked by green boxes. Two compounds were detected that could not be identified due to non-existent standards but their oxidative state can be estimated based on their ionized mass. X-oxoUDCA had the same mass as other bile acids with one ketone group and one hydroxyl group. The other unidentified compound had the same mass as UDCA and therefore it is likely to be an isoform with a 3β conformation (isoUDCA). The retention times for these compounds was unique and therefore could not be identified further. Concentration values of the unknown BAs could only be estimated for this reason. Error bars represent the standard deviation of the mean of biological triplicates

For UDCA, 7α-dehydroxylation to LCA was observed only with amendment of ^13^C-CA (Fig. 5). None of the strains exhibited any detectable level of activity from cultures that included only UDCA. 14.92 µM of LCA as well as extremely low amounts of 3-oxoLCA (with a maximum of 0.12 µM at 32 hours) were detected with *C. scindens* ATCC 35704 when ^13^C-CA was included. An unknown oxidized form labelled X-oxoUDCA was detected with a maximum concentration of 2.41 µM after 32 hours (Fig. 5A). It is likely that this BA corresponds to 3-oxoUDCA (3-oxo-7β-hydroxy-5β-cholan-24-oic acid) as we can exclude 7-oxoLCA (the other product of oxidation of UDCA) (Fig. 1). Another BA with the same ionized mass as UDCA was detected at a maximum concentration of 4.24 µM after 24 hours. We propose that this could be an isoform of UDCA with the hydroxyl group of the C3 carbon in the β conformation (3β,7β-dihydroxy-5β-cholan-24-oic acid). However, the identity of these compounds remains unconfirmed due to the lack of standards. In the presence of ^13^C-CA, the 7-DH-ing activity of *C. scindens* VPI 12708 was comparable to that of the ATCC strain in the presence of ^13^C-CA, with 13.61 µM of LCA and 0.68 µM of 3-oxoLCA after 48 hours (Fig. 5B). X-oxoUDCA was also detected at very small concentrations around 0.5 µM from 12 hours until the end of the experiment. The potential isoform of UDCA was detected at up to 9.92 µM at the 24-hour time point. Following the same trend observed with the other primary BAs, *E. muris* SJ24 did not show any detectable activity with UDCA with or without ^13^C-CA. The chromatograms for the unknown bile acids and the standards used can be found in Supplementary Figure 2 and Supplementary Table 4 respectively.

**Fig. 6:**
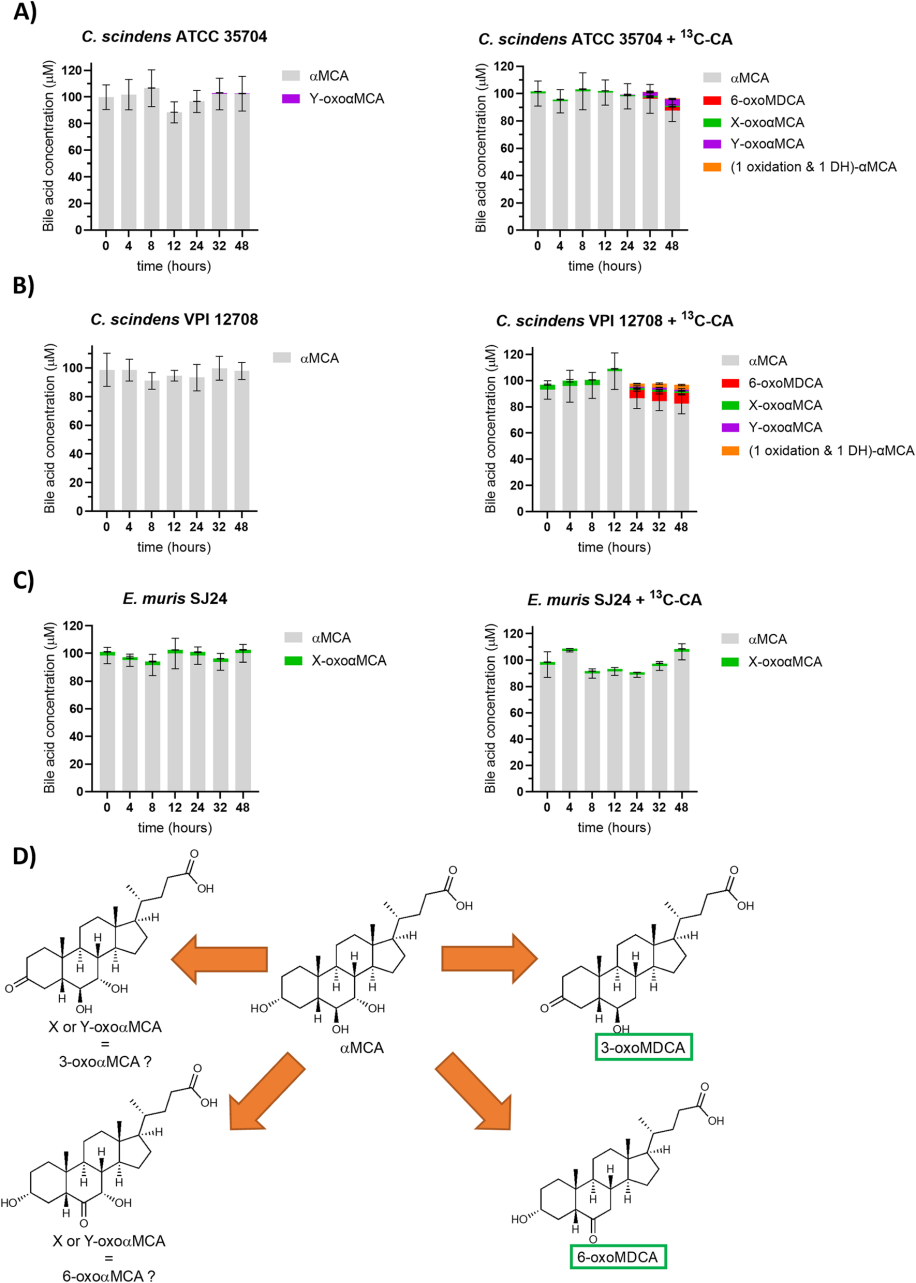
*In vitro* transformation of αMCA. The transformation of 100 µM of αMCA into secondary bile acids was tested with and without 100 µM of ^13^C-CA*.* **A** *Clostridium scindens* ATCC 35704, (**B**) *C. scindens* VPI 12708 and (**C**) *Extibacter muris* DSM 28561 (SJ24) were grown anaerobically in BHIS-S. Bile acids were extracted from suspended biomass. (**D**) Possible α-muricholic acid (αMCA) transformations, not necessarily all observed here. 7-dehydroxylated bile acids are marked by green boxes. Several compounds were detected that could not be identified due to non-existent standards but their oxidative state can be estimated based on their ionized mass. X- or Y- oxoαMCA had the same mass as other bile acids with one ketone group and two hydroxyl groups. The other unidentified compound had the same mass as secondary bile acids that have been dehydroxylated (-1 -OH), have one ketone group and one hydroxyl group. The retention times for these compounds did not correspond to that of any known standard. Concentration values of the unknown BAs could only be semi-quantitative for this reason. Error bars represent the standard deviation of the mean of biological triplicates

As expected, neither *C. scindens* strain nor *E. muris* were capable of αMCA 7α-dehydroxylation in the absence of ^13^C-CA (Fig. 6). *C. scindens* ATCC 35704 only produced minute amounts of an unknown oxo form of αMCA (labelled Y-oxoαMCA) (0.85 µM at 32 hours). In the *C. scindens* ATCC 35704 culture amended with ^13^C-CA, 6-oxoMDCA was detected at 2.7 µM after 48 hours (Fig. 6A). This secondary bile acid has been 7α-DH-ed but also the hydroxyl at C6 oxidized. Moreover, several intermediates for which standards are unavailable were also detected after 48 hours. These were unknown oxidized forms of αMCA (labelled X- and Y- oxoαMCA) at concentrations not exceeding 5 µM each. A third unknown BA was detected (albeit at very low concentrations, 0.41 µM at 32 hours) with the same mass as 6-oxoMDCA, suggesting that it is an MCA species with one oxidation and one dehydroxylation. This would indicate the production of another 7α-dehydroxylated form of αMCA *in vitro* (Figs. 1 and 6A). As for the ATCC 35704 strain, *C. scindens* VPI 12708 exhibited an increase in the quantity of products from αMCA transformation in the presence of ^13^C-CA relative to its absence (Fig. 6B). This includes the 7-DH-ed BA 6-oxoMDCA that reached a concentration of 8.18 µM after 48 hours and the X- and Y- αMCA forms that were detected at maximum concentrations of 4.22 µM (4 hours) and 1.69 µM (32 hours), respectively. The aforementioned αMCA-derived bile acid with one ketone group and one dehydroxylation was also detected at a maximum concentration of 3.49 µM after 48 hours (Fig. 6B). Surprisingly, *E. muris* SJ24 did not exhibit any observable 7-DH-ing activity with or without ^13^C-CA. Nevertheless, a small amount of X-oxoαMCA was detected at all time points, with a stable concentration at around 2.4 µM without and 1.9 µM with ^13^C-CA (Fig. 6C). The results for βMCA were very similar to those for αMCA and are discussed in further detail in the supplementary information.

The concentration of ^13^C-CA was also measured over time to ascertain that CA was being metabolized. It was observed to decrease until it disappeared after 48 hours in the *C. scindens* strains except in the presence of CDCA, for which the concentration decreased slowly over time. We attribute this observation to the toxicity of CDCA at that concentration [18]. On the other hand, the concentration of ^13^C-CA in *E. muris* remained stable over time and in all conditions, confirming lack of transformation (Supplementary Figure 3).

### *bai* gene expression in the presence of bile acids

To assess the impact of CA or other primary BAs on the expression of *bai* genes, the relative expression of *baiCD*, *baiE* and *baiJ* (an accessory gene to the operon) were measured. Gene expression was normalized using at least three reference genes and was calculated relative to the expression levels in a control group without BAs. The *E. muris* strains (SJ24, DSM 28561) has a truncated *baiJ* gene [22] but we deemed it worthy of investigation here. Additionally, *baiO* was also analysed for *E. muris* SJ24 as an alternative accessory *bai* gene [39].

**Fig. 7:**
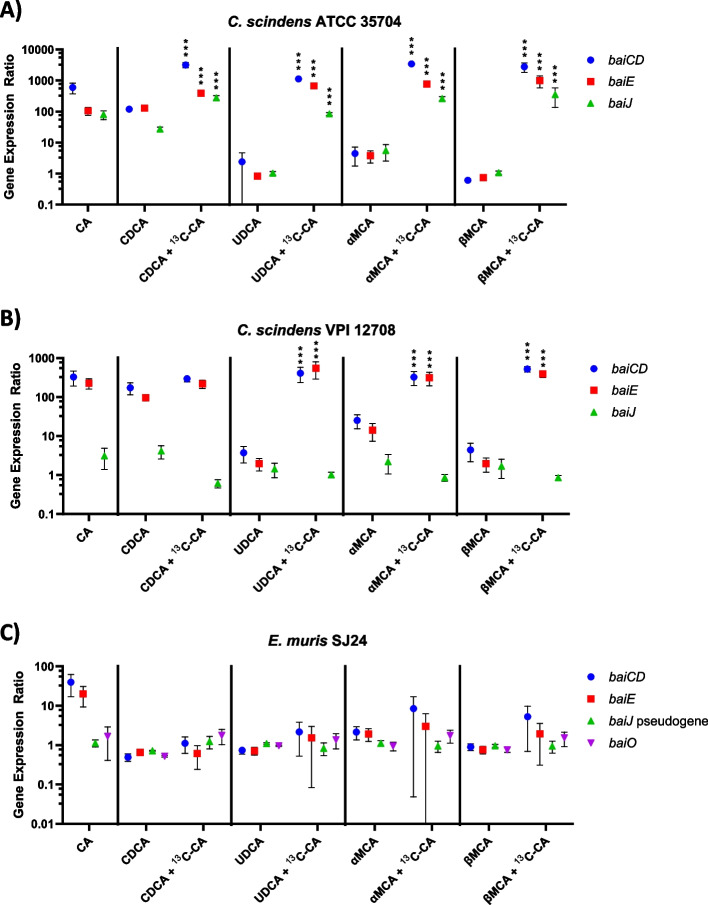
*bai* gene expression in the presence of CA (first panel) or in that of various primary BAs either as a single BA or with ^13^C-CA. Data for the CA-only condition correspond to the pooled expression results from the two sets of experiments, as described in the text. The expression (normalized to at least three reference genes) is relative to the no BA condition including an equivalent volume of solvent (ethanol). (**A**) *Clostridium scindens* ATCC 35704, (**B**) *C. scindens* VPI 12708 and (**C**) *Extibacter muris* DSM 28561 (SJ24) gene expression of *baiCD*, *baiE* (part of the *bai* operon), and accessory genes *baiJ* and *baiO* (only in *E. muris* DSM SJ24) was measured. CA, UDCA, αMCA, βMCA and ^13^C-CA were used at 100 µM, CDCA was used at 200 µM. A detailed view of the *C. scindens* VPI 12708 gene expression in presence of CDCA with or without ^13^C-CA is found in Supplementary Figure 4. Coloured dots represent the average and error bars represent the standard deviation of 12 replicates. Some error bars may look elongated due to the logarithmic scale of the Y axis. (***) indicates a *p*-value < 0.001 in a linear model analysis comparing the BAs with or without ^13^C-CA for each *bai* gene

Results show that the expression of *bai* operon genes in both *C. scindens* strains was highly upregulated in response to exposure to CA or to CDCA but not to the other BAs (Fig. 7).

For *C. scindens* ATCC 35704, the three genes tested were highly upregulated when ^13^C-CA was present along with another BA (CDCA, UDCA, αMCA, or βMCA) (Fig. 7A). In the CDCA dataset, statistically significant differences relative to the single BA condition were observed as all genes were slightly more upregulated in the presence of ^13^C-CA (*p*-value < 0.001 linear model), but *baiCD* was more so than the other genes (Fig. 7A). Most interestingly, UDCA, αMCA, or βMCA did not activate the expression of *bai* genes on their own, consistent with the lack of 7-DH-ing activity with these BA substrates alone (Fig. 7A).

A similar pattern was observed for *C. scindens* VPI 12708 but with the significant difference that *baiJ* was not upregulated under any conditions (Fig. 7B) (*p*-value < 0.001 linear model). In the CDCA dataset, the addition of ^13^C-CA had an upregulatory effect if assessed with a paired Wilcoxon test (Supplementary Figure 4) when compared to CDCA alone, but this effect was not found to be significant when using the linear statistical model displayed in Fig. 7. Similar to the case of *C. scindens* ATCC 35704, ^13^C-CA amendment had a dramatic effect on the expression levels of *baiCD* and *baiE* in the presence of UDCA, αMCA, or βMCA, with upregulation reaching the expression levels observed with CA or CDCA alone (Fig. 7B).

*E. muris* SJ24 showed a slight upregulation of *baiCD* and *baiE* in the CA dataset but it was not significant and did not occur in any of the other conditions (Fig. 7C), consistent with its very poor 7-DH-ing activity *in vitro* (Fig. 3). In fact, the increased *baiCD* and *baiE* gene expression ratio observed in the CA group was probably caused by a single biological replicate that had a higher expression level than the others.

Thus, CA had a large effect on *bai* expression, but in a strain-specific manner. Genes of the *bai* operon in the two *C. scindens* strains (ATCC 35704 and VPI 12708) exhibited a similar response to CA amendment but the accessory *baiJ* differed in its response. It was upregulated in strain ATCC 35704 but not in strain VPI 12708. In contrast, CA had no significant effect on the expression of any of the *bai* genes considered in *E. muris* SJ24.

The *rhaS*_1 gene (HDCHBGLK_01429) is immediately upstream of the *bai* operon promoter on the opposite strand and has been proposed as bile acid-regulatory A (*barA*) due to its potential implication in *bai* regulation [7]. The expression of *rhaS1* and *rhaS2* (a copy of *rhaS1* elsewhere in the genome) was shown to have background levels in the presence of all BAs (Supplementary Figure 5). This was tested in *C. scindens* ATCC 35704 without the amendment of ^13^C-CA. Results indicate that *rhaS* is not upregulated by the presence of the BAs tested.

Thus, the question remains about the conditions propitious for *bai* gene expression and robust 7-DH-ion in *E. muris* SJ24. We hypothesized that other mouse-specific BAs may be key regulators.

### *bai* gene regulation by other BAs in *E. muris* SJ24

**Fig. 8:**
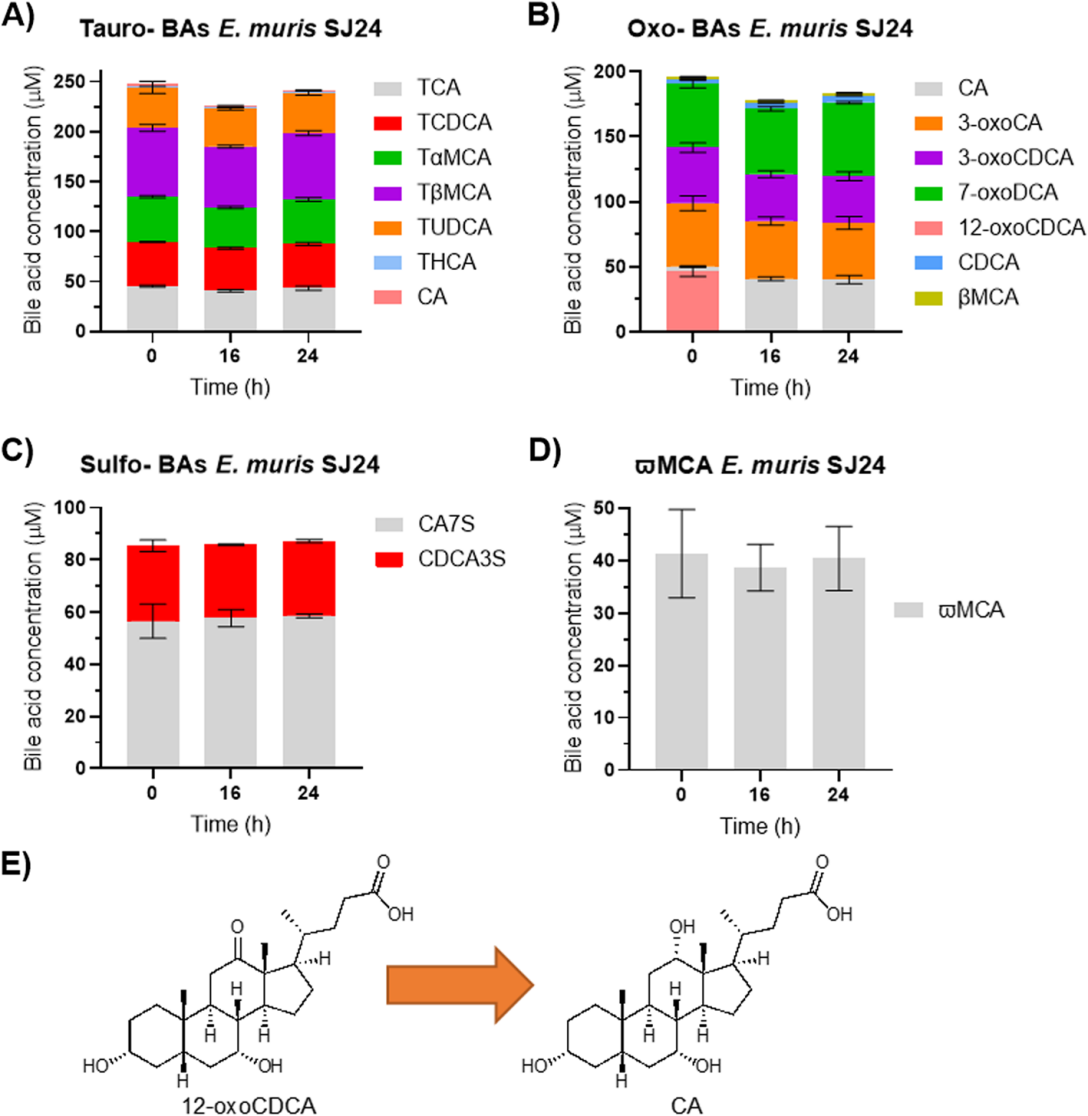
*In vitro* transformation of BA cocktails by *E. muris* DSM SJ24. The transformation of several BAs at 50 µM by *E. muris* DSM 28561 (SJ24) was tested anaerobically in 25 mL of BHIS-S. Different cocktails were prepared based on their similarity. (**A**) Tauro-conjugated BAs, (**B**) Oxidized BAs, (**C**) Sulfonated BAs or (**D**) the secondary BA ωMCA which is present in the murine BA pool. Three time points were taken for BA extraction from suspended biomass. An additional sample of 1 mL was taken at 16 hours for RNA extraction and RT-qPCR analysis. Minute concentrations (<2 µM) of CA (**A**) and βMCA (**B**) were detected, most likely as impurities from the standards used as they were detected from time 0 and concentrations remained stable. (**E**) Represents the single transformation detected in (**B**) where 12-oxoCDCA was entirely reduced to CA. Error bars represent the standard deviation of the mean of biological replicates

Because the presence of CA or other primary BAs did not affect *bai* gene expression in *E. muris* SJ24, we tested four BA cocktails to probe whether other BAs commonly found in the BA pool could promote *bai* expression. The BA pool was divided into four cocktails: tauro-conjugated BAs, oxidized BAs, sulfonated BAs, or ωMCA. The addition of these BAs mixtures to *E. muris* SJ24 did not yield the production of any detectable secondary BAs (Fig. 8). A small CA concentration (<2 µM) was detected with the tauro-BA cocktail (Fig. 8A) but this was likely the result of the presence of CA as an impurity in the TCA standard, as it was also detected at time 0. In the oxidized BA cocktail, 12-oxoCDCA was almost fully reduced to CA after 16 hours (Fig. 8B). Small quantities of CDCA and βMCA were detected, while both are likely to be impurities from the standards used (detected at time 0), it is worth highlighting that the concentration of CDCA increased from an average of 2.74 µM (time 0) to 5.35 µM (time 24), meanwhile, the concentration of βMCA remained stable around 2 µM. No reduction of 3-oxo forms was detected, likely due to the absence of *baiA2* [24]*.* Finally, neither sulfonated BAs nor ωMCA were transformed by *E. muris* SJ24 in any way (Fig. 8C-D). Therefore, we considered unlikely that these other tested BAs could upregulate *bai* expression without being substrates for 7-DH-ion.

The expression of *baiCD*, *baiE*, the pseudogene *baiJ* and *baiO* was nonetheless measured in the BA cocktail experiments and compared with a CA-only reference group. Given the lack of 7-DH-ion of the BAs within the cocktails, it is not surprising that no significant upregulation was observed in any of the BA cocktail groups when compared to the CA control. (Supplementary Figure 6A).

### Bile acid 7-DH-ion by *E. muris* SJ24 in the presence of mouse cecal content

**Fig. 9:**
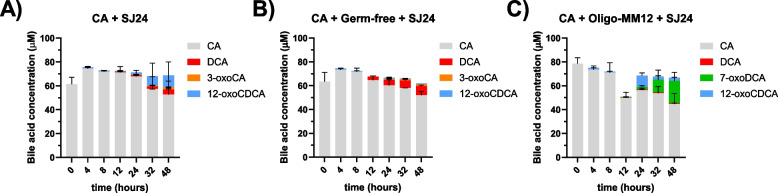
*In vitro* 7-DH-ion of CA by *E. muris* SJ24. The transformation of 100 µM of CA into secondary BAs by strain SJ24 was tested with and without the amendment of cecal content. Control group with only CA (**A**) was used as intra-assay reference vs. (**B**) cecal content from germ-free mice and (**C**) cecal content from sDMDMm2 mice. 25 mg of cecal content were added to 25 mL of BHIS-S. The mass imbalance between the added CA (100 µM) and the measured (approx. 80 µM) can be attributed to the presence of CoA- forms that cannot currently be quantified due to lack of standards. Error bars represent the standard deviation of biological triplicates. Data from the control groups of this experiment can be found in Supplementary Figure 7

CA 7-DH-ion by *E. muris* SJ24 was investigated in the presence of cecal content from either germ-free mice or a stable gnotobiotic murine model, Oligo-Mouse-Microbiota (Oligo-MM12) [40] in order to further investigate potential non-BA triggers for 7-DH-ion. A significant fraction of CA was conjugated with Co-enzyme A (CoA) and therefore could not be rigorously identified or quantified, as there are no standards for CoA- forms. In the controls (no cecal content), the DCA concentration averaged 4.26 µM after 48 hours which corresponded to the transformation of approximately 7% of the initial CA (Fig. 9A). The amendment of cecal content from germ-free mice increased the DCA produced to 7.6 µM which corresponded to 12% of the initial CA (Fig. 9B). Finally, the addition of cecal content from Oligo-MM12 mice produced only 0.67 µM of DCA but 18.6 µM of 7-oxoDCA (Fig. 9C). In all conditions, DCA was detected after 12 hours of incubation and gradually increased. 12-oxoCDCA was detected in all conditions, while 3-oxoCA was only found in the no cecal content and germ-free groups (Fig. 9). The control groups of cecal content without *E. muris* SJ24 showed no change in CA concentration other than the potential conjugation with Co-A by the Oligo-MM12 mouse case (Supplementary Figure 7).

Despite the measurable impact on 7-DH-ion by the addition of germ-free mouse cecal content of CA 7-DH-ion, it was not sufficient to significantly upregulate *bai* expression when compared to the CA-only reference group (Supplementary Figure 6B). In both assays, the gene expression ratio of *bai* genes was never above 3.

### *E. muris* SJ24 *in vivo* 7-DH-ion and *bai* gene expression

The ability of *E. muris* strain DSM 28560 (JM40) to 7-DH-ate *in vivo* has been previously documented [22]. Here, colonisation of Oligo-MM12 gnotobiotic mice was performed with the DSM 28561 strain (*E. muris* SJ24) to confirm 7-DH-ion *in vivo* and assess *bai* gene expression. The bile acid composition confirms active 7-DH-ion *in vivo* in Oligo-MM12 mice. Indeed, DCA, LCA and MDCA, were exclusively identified in the sDMDMm2 + *E. muris* SJ24 group (Supplementary Figure 8).

As above, expression of *baiCD*, *baiE,* the pseudogene *baiJ* and *baiO* was assessed, but no relative quantification was performed due to the absence of *bai* genes in the Oligo-MM12 control mice (not colonized with *E. muris* SJ24). Transcripts of all *bai* genes were detected in the Oligo-MM12 mice colonized with *E. muris* SJ24. We observed non-specific amplification of transcripts in the Oligo-MM12 control mice with *bai* primers, but at a very low level compared to the *E. muris-*colonized Oligo-MM12 mice (Supplementary Table 2). Considering the evidence of secondary BAs produced in the colonized mice (Supplementary Figure 8), we conclude that *E. muris* expresses *bai* genes *in vivo*.

## Discussion

Bile acid chemistry is a relevant field in human and veterinary medicine not only because of the BA’s detergent function during digestion but also for the wide range of roles related to host physiology and homeostasis [41, 42].

The *bai* operon was originally described in *C. scindens* VPI 12708 in 1990 and this strain has become the reference for biochemical studies of the 7-DH-ion pathway [23, 38, 43]. The operon (*baiBCDEA2FGHI*) encodes one CoA ligase (*baiB*), two oxidoreductases (*baiCD* and *baiH*), a 7-dehydratase (*baiE*), a 3α-HSDH (*baiA2*), a CoA transferase (*baiF*), a transporter (*baiG*), and a putative ketosteroid isomerase (*baiI*) (Fig. 2), but not all genes are required for 7-DH-ion [24]. Indeed, it was shown that CA 7-DH-ion requires 6 proteins (BaiB, BaiA2, BaiCD, BaiE, BaiF, BaiH) in *C. scindens* VPI 12708 [24] and CDCA 7-DH-ion 5 proteins (BaiB, BaiA2, BaiCD, BaiE, BaiJ) [19]. Expectedly from such a complex operon, comparative genomics have highlighted significant differences in the *bai* operon amongst 7-DH-ing strains [31, 38]. Moreover, accessory *bai* genes have also been described across multiple strains [38, 43]. These accessory genes often cluster in two operons *baiJKL* and *baiNO* which are not always complete; *C. scindens* ATCC 35704 only has *baiJ* (urocanate reductase) [35] while the VPI 12708 strain has the full *baiJKL* set [35] (Fig. 2). From genome analysis, *E. muris* SJ24 has fragments of *baiJKL* as pseudogenes. The exact role of these genes has yet to be defined although there is novel evidence of the role of BaiJ [19, 44].

Out of all primary BAs, only CA has been consistently reported to be 7-DH-ed *in vitro*. In contrast, CDCA is poorly 7-DH-ed and other murine primary BAs (αMCA, βMCA or UDCA) are not at all [18, 22, 45–48]. In the present work, we confirm that the two *C. scindens* human isolates upregulated the expression of *bai* operon genes (i.e., *baiCD* and *baiE*) in response to CA (Fig. 7), but found that it only upregulated that of *baiJ* in the ATCC 35704 strain, not the VPI 12708 strain*.* This accessory gene is annotated as an urocanate reductase and a member of the oxidoreductase family. The current CA 7-DH-ion pathway would suggest that this gene is not involved in that process in strain VPI 12708 [24], which is consistent with the low level of expression observed here (Fig. 7B). Conversely, its high level of upregulation in strain ATCC 35704 matches previously published data [19, 35] and suggests that *baiJ* may play a role in the 7-DH-ion of CA or other BAs (e.g., CDCA [19, 44]) in that strain. Finally, *E. muris* SJ24, the murine isolate, barely registered any *bai* gene expression response to CA or any other BA tested.

*C. scindens bai* gene expression was upregulated by both CDCA and CA (Fig. 7). On average, amendment with ^13^C-CA increased expression 13.2-fold in ATCC 35704 and 1.98-fold in VPI 12708 (except for *baiJ* for the latter) compared to CDCA alone. With the amendment, the concentration of the products of CDCA 7-DH-ion rose from 1% to 5% in strain ATCC 35704 and from 1% to 23% in strain VPI 12708 (Fig. 4). Thus, a large increase in expression for strain ATCC 35704 did not translate to a large increase in 7-DH-ion, suggesting that *bai* gene expression is not the bottleneck for efficient CDCA 7-DH-ion in ATCC 35704. Moreover, the modest VPI 12708 *bai* expression increase does not explain the large increase in CDCA 7-DH-ion with ^13^C-CA. We hypothesise that the impact of CA on CDCA 7-DH-ion is not solely via *bai* gene expression but may involve unknown genetic components or CA-derived co-factors.

UDCA is the 7β isomer of CDCA and is reported to have significant therapeutic properties [2, 7, 49]. In humans, UDCA is synthesized from CDCA by gut microbes containing 7β-HSDHs [7, 27, 50, 51] and is thus, a secondary BA; in mice, it is produced by the liver, thus, it is a primary BA, and is used as a precursor for βMCA [15, 16]. Nevertheless, colonisation of mice lacking 7-dehydroxylating bacteria with 7-DH-ing bacteria increases UDCA levels, implying that the gut microbiome also plays a significant role in UDCA production in mice [14]. Despite the importance of this BA for medical applications, little is known about the capacity of bacteria to 7β-dehydroxylate UDCA *in vitro.* Previous work has reported no transformation by *C. scindens* strain ATCC 35704 [18]. In accordance with our hypothesis, amendment of ^13^C-CA not only greatly upregulated *bai* gene expression (Fig. 7B) but also provided evidence of UDCA 7β-dehydroxylation as LCA was detected in the culture (Fig. 5). Indeed, the 7-DH-ed product amount increased in the presence of ^13^C-CA from 0 to 15 and 16% in *C. scindens* strains ATCC 35704 and VPI 12708, respectively. The absence of CDCA in the culture suggests that UDCA was not first epimerized to CDCA and subsequently 7α-dehydroxylated. Moreover, two unidentified compounds consistent with 7β-dehydroxylation were detected, providing further evidence of direct UDCA 7β-dehydroxylation and its associated unique set of intermediates. The oxidized intermediate is very likely to be 3-oxoUDCA (3-oxo-7β-hydroxy-5β-cholan-24-oic acid), because, based on the mass, the only alternative would have been 7-oxoLCA, which can be excluded as it is one the standards in our collection (Fig. 5). The other unknown compound has the same mass as UDCA albeit a different retention time, suggesting this could be an iso- form of UDCA. It follows that this would be 3β-UDCA, tentatively named isoUDCA (3β,7β-dihydroxy-5β-cholan-24-oic acid). Isoforms are well known in the BA pool and particularly in CDCA-related intermediates [18, 24, 36, 52] so it is likely that UDCA follows a similar pattern. Despite the production of unknown intermediates, the activity response to ^13^C-CA suggests that UDCA 7β-dehydroxylation uses some of the same Bai machinery as CDCA 7α-dehydroxylation.

7-DH-ion activity was uncovered for αMCA and βMCA, for the first time and it yielded several intermediate BAs that we were not able to fully characterize due to the lack of appropriate standards. All human and mouse BAs share a backbone of four rings (Fig. 1), this makes mass fractionation in the mass spectrometer unsuitable for identification. Therefore, we currently rely on comparison of ionized mass and retention time to standards. However, several assumptions can be made to speculate what these compounds could be. MCAs could be oxidized at the C-3, C-6 or C-7 position (Figs. 1 and 6D)*.* A C-7 oxidation would yield 7-oxoMDCA regardless of the primary MCA. Meanwhile, other oxidations could be differentiated by the α or β conformation of the C-7 hydroxyl. It is possible that one of the intermediates that we detected was 7-oxoMDCA, but none shared retention times across MCAs, meaning that different intermediates were produced for each of the two MCA substrates (Supplementary Figure 2). Considering that distinct oxidized intermediates were detected for the two MCAs, we hypothesize that those are the 3-oxo and 6-oxo forms of αMCA and βMCA (*3-oxoαMCA*: 3-oxo-6β,7α-dihydroxy-5β-cholan-24-oic acid; *6-oxoαMCA*: 6-oxo-3α,7α-dihydroxy-5β-cholan-24-oic acid; *3-oxoβMCA*: 3-oxo-6β,7β-dihydroxy-5β-cholan-24-oic acid; and *6-oxoβMCA*: 6-oxo-3α,7β-dihydroxy-5β-cholan-24-oic acid). A third intermediate was also detected from both MCAs, with the ionized mass corresponding to secondary BAs with one dehydroxylation and a ketone group (e.g., 7-oxoLCA) (Fig. 6 & Supplementary Figure 9). Three options are plausible: 1) A dehydroxylation at the C-3 position. This would yield a novel family of BAs with a 6β- and 7α/β- hydroxyls, one of which is oxidized to a ketone. This option is highly unlikely as it would have been identified previously by the multiple studies investigating the murine BA pool [15, 16, 53–56]. 2) An oxidation paired with a 6-dehydroxylation would yield 3-oxoCDCA, 7-oxoLCA, or 3-oxoUDCA. 3-oxoCDCA and 7-oxoLCA were included as standards in our analysis (Supplementary Table 3) and would have been detected if present. Meanwhile, the retention time of this compound is distinct from that of the compound proposed to be 3-oxoUDCA from the transformation of UDCA (see above and Supplementary Figure 2). Thus, this is not likely to be 3-oxoUDCA. 3) A 7-dehydroxylation could allow for ketone groups at the C-3 and C-6 positions. 6-oxoMDCA was available as a standard but, the second option, 3-oxoMDCA (3-oxo-6β-hydroxy-5β-cholan-24-oic acid), was not. It is therefore possible that this compound corresponds to 3-oxoMDCA, but this remains to be confirmed.

The mouse BA pool is significantly more diverse than that of humans due to the primary production of muricholic acids and of UDCA. The murine secondary BA pool includes DCA and LCA but also MDCA and its 6α counterpart, hyodeoxycholic acid (HDCA). Furthermore, mice can rehydroxylate TDCA back into TCA in the liver [15] which magnifies the differences between mouse and human BA pools. In general, the secondary BAs derived from muricholic acids seem to be in low abundance in the gut, hinting at the difficulty of 7-DH-ing these BAs. This is perhaps the reason why primary BAs such as βMCA are highly abundant in the mouse BA pool [57]. While we initially hypothesized that the lack of 7-DH-ion activity for αMCA and βMCA was due to the lack of *bai* gene expression, our data show that even *bai* gene expression in the *C. scindens* strains is insufficient for the production of MDCA [22], and results instead in the detection of potential oxidized versions of MDCA (Fig. 6 and Supplementary Figure 9).

As reported above, the murine strain *E. muris* SJ24 has shown no significant *bai* upregulation nor 7-DH-ion *in vitro* either in the presence or absence of ^13^C-CA. To investigate the underlying reasons for this lack of activity, we considered three additional conditions: (a) various BA mixtures, to determine whether *bai* gene expression was controlled by another (or several other) murine BAs; (b) *in vitro* in the presence of germ-free or Oligo-MM12 cecal content to ascertain whether the presence of other gut bacteria or signalling molecules from the host itself induced 7-DH-ion; or (c) in the Oligo-MM12 environment, to confirm the activity of strain SJ24 *in vivo*.

The *in vivo* condition exhibited *bai* expression (Supplementary Table 2) and 7-DH-ed BAs were detected in the BA pool from the same samples (Supplementary Figure 8). Thus, *E. muris* SJ24 is capable of *in vivo* 7-DH-ion, although its *bai* machinery may require additional genes that have not been identified in this study to efficiently 7-DH-ate *in vitro*. However, the exact trigger for *in vivo* levels of 7-DH-ion from *E. muris* remains elusive at this point.

To elucidate that question, we tested all the BAs detected in the Oligo-MM12 environment and found no evidence of 7-DH-ing activity (Fig. 8 and Supplementary Figure 6A), excluding the possibility that non-CA BA triggered *bai* gene expression in *E. muris*.

However, when strain *E. muris* SJ24 was grown in the presence of cecal content from germ-free mice, its 7-DH-ing activity increased (Fig. 9) despite *bai* gene expression not increasing significantly (Supplementary Figure 6B). Indeed, the amendment of cecal content from germ-free mice (Fig. 9B) resulted in an almost two-fold increase from 7 to 13% of 7-DH-ed products, coupled with a decrease of the abundance of the 12-oxoCDCA intermediate. Interestingly, the co-cultivation of *E. muris* with the non-sterile cecal-content from Oligo-MM12 mice produced high amounts of 7-oxoDCA (Fig. 9C) while CA was not transformed by the same cecal content in the absence of strain SJ24 (Supplementary Figure 7). This could suggest an interaction between *E. muris* and the gnotobiotic community in which the former would promote the 7-oxidation of CA by the latter, known to harbor 7-HSDHs [58]. This interaction appears to be exclusive to the *in vitro* environment since the *in vivo* BA data show lower concentrations of 7-oxoDCA than DCA (Supplementary Figure 8).

The evidence presented in this study shows that the role of the host, presumably through signalling, is a critical element for effective 7-DH-ion by *E. muris* and the regulatory mechanisms of this secondary BA transformation is dramatically different amongst bacterial species. The results also highlight the significant differences in 7-DH-ion between human and mouse isolates but also between the *in vitro* and *in vivo* environments.

To further highlight the differences, the human isolates showed marginal activity for αMCA and βMCA upon addition of ^13^C-CA. 7-DH-ed forms such as 6-oxoMDCA were detected (Fig. 6 & Supplementary Figure 9) but the full 7-dehydroxylation to MDCA was not observed. These data add more evidence that other elements besides the *bai* operon (and *baiJ*) might be needed to 7-dehydroxylate MCAs to MDCA. Perhaps, a missing 6β-HSDH gene would be required for complete 7-DH-ion. The apparent simplicity of 7-DH-ion regulation from human isolates compared to that of *E. muris* could be due to the more diverse diet of humans as compared to mice. It has been observed that a less diverse diet can overstimulate the BA pool in humans and increase the incidence of colorectal cancer [59]. The natural mouse diet is less diverse than that of humans and their initial lactation period (a monotrophic diet) plays a much stronger role in the mouse lifespan [60]. In these circumstances, a strong regulation of 7-DH-ion might be an important mechanism to prevent BA pool unbalances. Nevertheless, much more data on the 7-DH-ion mechanisms of various strains with particular focus on isolates from the mouse and other animal models is required to investigate the potential differences in 7-DH-ion regulation.

## Conclusion

The findings presented here are fourfold. First, we demonstrated that the previously reported [35, 36] strong upregulation of *bai* genes by CA increases the extent of 7-DH-ion of other primary BAs. This was particularly true for UDCA which has been reported to be converted to LCA *in vitro* for the first time.

Secondly, the upregulation of *bai* genes exhibited strain-specific differences. *C. scindens* ATCC 35704 upregulated the *bai* operon genes *baiCD* and *baiE* as well as the *bai* accessory gene *baiJ*. While for *C. scindens* VPI 12708, it upregulated the *bai* operon genes, but *baiJ* expression was found to be at a background level. This is consistent with the lack of involvement of *baiJ* in 7-DH-ion in strain VPI 12708 [24]. *E. muris* SJ24 was the third bacterium tested, a murine isolate with *in vivo* 7-DH-ing capabilities (Supplementary Figure 8). Strain SJ24 showed weak *in vitro* 7-DH-ion of CA and no upregulation of any of the *bai* genes tested. The activity of this strain was promoted by the addition of germ-free cecal content but not cecal content from gnotobiotic Oligo-MM12 colonized mice. This result suggests that a host factor is required for efficient 7-DH-ing activity by strain SJ24 but that the presence of a minimal microbiome (Oligo-MM12 consortium) inhibits this activity potentially due to the promotion of 7α-HSDH activity from the microbiome *in vitro*. Unravelling the controls on BA 7-DH-ion by *E. muris* requires further investigation.

Thirdly, *C. scindens* human isolates can partially 7-dehydroxylate MCAs, leading to the formation of oxidized MDCA at the C-6 position. Therefore, an enzyme capable of reducing this compound, e.g., a 6β-HSDH, is required to achieve the end point of 7-DH-ion that is observed *in vivo*, namely MDCA. To date, no such protein has been identified in any microorganism.

Finally, the mechanism of 7-DH-ion regulation appears to differ significantly between the murine- and human-derived strains tested here, which could be due to the nature of the host. Human isolates showcase a system governed by CA, while the presently investigated murine isolate appears to utilise a BA-independent signal present in the lumen.

In conclusion, these data provide novel insights into the intricacies of 7-DH-ion and the significant differences amongst 7-DH-ing bacteria. The CA-dependent response can be attributed to the abundance of this compound in the BA pool of humans. However, the regulatory factor for *E. muris* activity remains elusive despite evidence suggesting that it is host derived. Moreover, multiple novel BAs were observed, and their identity surmised. Further work with these and other strains is required to investigate the 7-DH-ion pathway of CDCA, αMCA, βMCA and UDCA as well as to explore the strain-specific differences regarding the 7-DH-ion pathway of CA.

## Materials and methods

### Bacterial strains and growth conditions

The strains used were *Clostridium scindens* ATCC 35704, *Clostridium scindens* VPI 12708 and *Extibacter muris* DSM 28561 (SJ24), this strain was chosen instead of *E. muris* DSM 28560 (JM40) due to its ability to grow faster *in vitro* (24h vs 48h, data not shown). Bacteria were grown anoxically in Brain Heart Infusion Supplement – Salts (BHI-S) medium, consisting of 37g BHI, 1g L-cysteine, 5g yeast extract, 2.5g fructose, 50mL salts solution (0.2g CaCl_2_, 0.2g MgSO_4_, 1g K_2_HPO_4_, 1g KH_2_PO_4_, 10g NaHCO_3_ and 2g NaCl per L of ddH_2_O) per L ddH_2_O. The salts solution and media were sterilised by autoclaving. Static growth was carried out at 37°C in an anoxic chamber (Coy Laboratory Products, 95% N_2_, 5% H_2_). A pre-inoculum was prepared from glycerol stocks (using BHIS-S) before inoculating 25 mL of BHIS-S in Falcon tubes at a starting OD_600_ of 0.05. Results of the growth curves for the experiments can be found in the Supplementary Information (Supplementary Figure 10).

### *In vitro* 7-dehydroxylation assays

Bacteria were grown in presence of BAs: CA (100 µM), CDCA (200 µM), αMCA (100 µM), βMCA (100 µM) and UDCA (100 µM) or the same volume of ethanol (solvent control) (e.g,. 250 µL in a 25 mL culture). A sterile control (media with ethanol solvent) was also included. CA amendment was performed by adding an additional 100 µM of ^13^C-CA to the other BAs at time 0. ^13^C-CA was chosen so its transformation products could be separated from those of the other primary BAs during the quantification process. All experiments without ^13^C-CA were conducted at the same time while all the amended ones were run simultaneously at a different time. The amendment of only 100 µM CA was done in both experiments, this allows for cross-comparison of the results. Growth was monitored by periodically measuring the OD_600_. During the main time points (0, 4, 8, 12, 24, 32 and 48 hours), 1 mL samples were collected for BA extraction in a 2 mL bead-beating resistant tube and stored at -80°C until processing. All conditions were performed in triplicates.

Four BA cocktails were prepared for the assays with *E. muris* SJ24 based on the BAs often present in a meaningful amount within the BA pool of mice. All BAs within the cocktails were at 50 µM. The Tauro- conjugated cocktail included TCA, TCDCA, TαMCA, TβMCA, TUDCA and THCA. The Oxo- cocktail included 3-oxoCA, 3-oxoCDCA, 7-oxoDCA and 12-oxoCDCA. The Sulfo- cocktail included CA7S and CDCA3S. Finally, the last group was only comprised of 50 µM of ωMCA. All BAs were diluted in ethanol or methanol depending on their solubility. Three time points were collected (0, 16 and 24 hours).

Furthermore, *E. muris* SJ24 was also amended with cecal content to test its implications over 7-DH-ion *in vitro*. For this assay, 25 mg of freeze-dried cecal content from germ-free mice or 25 mg of frozen content with 5% glycerol (v/v) from sDMDMm2 mice were added to 25 mL of BHIS-S with 100 µM CA. The control conditions for this experiment were a group with 100 µM CA but no additional cecal content and two more groups with each respective type of cecal content but no *E. muris* SJ24. Seven time points were taken (0, 4, 8, 12, 24, 32 and 48). RNA sample collection was particularly early for this experiment (4-hour time point) due to a faster-than-usual growth (Supplementary Figure 10).

### Bile acid extraction

Samples were vacuum dried overnight (ON) in a Speedvac (Christ) at room temperature. Approximately 450 mg of 0.5 mm zirconium beads were added to the dried samples as well as 500 µL ice-cold alkaline acetonitrile (acetonitrile – 25% ammonia 4:1 v/v) and 100 µL of ISTD solution (CA-d_4_, CDCA-d_4_, TCA-d_4_, TUDCA-d_4_, DCA-d_4_ and LCA-d_4_, each at 100 µM in methanol). Samples were homogenized in a Precellys 24 Tissue Homogenizer (Bertin Instruments, Montigny-le-Bretonneux, France) at 6500 rpm 3x 30” beat 30” rest. Samples were vortexed for 1 hour and centrifugated for 15 minutes at 16000 rcf at room temperature. Approximately 500 µL of suspension was carefully collected over the beads level and transferred into a new 1.5 mL epi tube which was then vacuum dried overnight. Finally, the samples were reconstituted in 1 mL of ammonium acetate [5mM] – methanol (50:50 v/v) and a 1:20 dilution with the same solvent was prepared in LC-MS glass vials, ready for injection.

### RNA extraction and reverse transcription

1 mL of sample was collected in a 15 mL falcon tube during the mid-log to late-log phase for the RNA extraction. The sample was stored with RNAprotect following the manufacturer protocol (Protocol 5 from RNAprotect Bacteria Reagent Handbook 01/2020, Qiagen) at -80°C until processed. All conditions were performed in triplicates. Lysis and RNA purifications were done using the RNeasy Mini Kit (Qiagen, Hilden, Germany). Bacterial lysis was performed following Protocol 5: Enzymatic Lysis, Proteinase K Digestion and Mechanical Disruption of Bacteria (RNAprotect Bacteria Reagent Handbook 01/2020, Qiagen), with 20 µL of proteinase K for each sample and the required volumes for a number of bacteria <7.5 x 10^8^. The cell lysis was performed using a Precellys 24 Tissue Homogenizer (Bertin Instruments, Montigny-le-Bretonneux, France) at 6500 rpm 3x 10 seconds beat 10 seconds rest. RNA purification was performed following Protocol 7: Purification of Total RNA from Bacterial Lysate using the RNeasy Mini Kit. Centrifugations were carried out at 15000 rcf except for the 2 min centrifugation which was done at 18000 rcf.

Purified RNA was further subject to a DNase treatment using the RQ DNase I (Promega, Madison, WI, USA) following the manufacturer protocol with small modifications: The final volume was adjusted to a 100 µL and incubation was extended to 1 hour at 37°C. The treated RNA was cleaned-up using the RNeasy Mini Kit (Qiagen, Hilden, Germany) following the RNA Clean-up protocol from the manufacturer (RNeasy Mini Handbook 10/2019) with the 2 min centrifugation done at 18000 rcf. Concentration and purity of RNA was measured with a NanoDrop One (Thermo Fisher Scientific, Waltham, MA, USA).

100 ng of RNA was reverse transcribed into cDNA using the GoScript™ Reverse Transcription Mix, Random Primers (Promega, Madison, WI, USA) following the manufacturer protocol. The process was done in duplicates with one group using water instead of the reaction buffer as a non-reverse transcription control (NRT).

### Reverse transcription quantitative PCR (RT-qPCR)

RT-qPCRs were prepared using the Myra liquid handling system (Bio Molecular Systems, software version 1.6.26) and performed using the Magnetic induction cycler (Mic) platform (Bio Molecular Systems, Upper Coomera, QLD, Australia) with the micPCR software (v2.10.5).

The list of primers used can be found in Supplementary Table 3. Samples were prepared with the SensiFAST SYBR No-ROX Kit (Meridian Bioscience, Cincinnati, OH, USA) at a final volume of 10 µL. All runs were performed with the following program, with small modifications: Initial hold at 95°C for 5 minutes with a cycle of 95°C for 5 seconds, 54.5°C for 20 seconds (54.1°C for *E. muris* SJ24) and 72°C for 9 seconds. 40 cycles were done for *C. scindens* ATCC 35704 and 50 for *C. scindens* VPI 12708 and *E. muris* SJ24. The melting curve, temperature control and acquisition settings were left as default. The quantification was done using three or more reference genes (Supplementary Table 3) based on their expression stability across conditions. NRTs as well as no template controls (NTCs) were included to check for residual DNA or contaminations. Four technical replicates were included for each biological replicate. Note that expression data presented in Fig. 7 for the CA-only condition (labelled CA) correspond to the pooled expression results (for the condition in which only 100 µM CA was added) from two sets of experiments.

### Liquid chromatography – mass spectrometry (LC-MS)

The quantitative method was performed on an Agilent ultrahigh-performance liquid chromatography 1290 series coupled in tandem to an Agilent 6530 Accurate-Mass Q-TOF mass spectrometer. The separation was done on a Zorbax Eclipse Plus C18 column (2.1 x 100mm, 1.8 µm) and a guard column Zorbax Eclipse Plus C18 (2.1 x 5mm, 1.8 µm) both provided by Agilent technologies (Santa Clara, CA, USA). The column compartment was kept heated at 50°C. Two different solutions were used as eluents: ammonium acetate [5mM] in water as mobile phase A and pure acetonitrile as mobile phase B. A constant flow of 0.4 mL/min was maintained over 26 minutes of run time with the following gradient (expressed in eluent B percentage): 0-5.5 min, constant 21.5% B; 5.5-6 min, 21.5-24.5% B; 6-10 min, 24.5-25% B; 10-10.5 min, 25-29% B; 10.5-14.5 min, isocratic 29% B; 14.5-15 min, 29-40% B; 15-18 min, 40-45% B; 18-20.5 min, 45-95% B; 20.5-23 min, constant 95% B; 23-23.1 min, 95-21.5% B; 23.10-26 min, isocratic 21.50% B. The system equilibration was implemented at the end of the gradient for 3 minutes in initial conditions. The autosampler temperature was maintained at 10°C and the injection volume was 5µL. The ionisation mode was operated in negative mode for the detection using the Dual AJS Jet stream ESI Assembly. The QTOF acquisition settings were configured in 4GHz high-resolution mode (resolution 17000 FWHM at m/z 1000), data storage in profile mode and the high-resolution full MS chromatograms were acquired over the range of m/z 100-1700 at a rate of 3 spectra/s. The mass spectrometer was calibrated in negative mode using ESI-L solution from Agilent technologies every 6 hours to maintain the best possible mass accuracy. Source parameters were setup as follows: drying gas flow, 8 L/min; gas temperature, 300°C; nebulizer pressure, 35psi; capillary voltage, 3500V; nozzle voltage, 1000V. Data were processed afterwards using the MassHunter Quantitative software and MassHunter Qualitative software to control the mass accuracy for each run. In the quantitative method, 42 bile acids were quantified by calibration curves (Supplementary Table 4). The quantification was corrected by addition of internal standards in all samples and calibration levels. Extracted ion chromatograms were generated using a retention time window of ± 1.5 min and a mass extraction window of ± 30ppm around the theoretical mass of the targeted bile acid. Unknown BAs were identified when found within the retention time window of a standard with the same ionized mass. Approximate quantification of these unknown BAs was done by using the nearest standard (by retention time) with the same ionized mass. No standards are available for these unknown BAs showed in Supplementary Figure 2.

### *In vivo* colonisation with *E. muris* SJ24

A cohort of nine Oligo-MM12 [61] mice were used. Four mice were dedicated to an uncolonized control group and the remaining five were colonized with *E. muris* DSM 28561 (SJ24). All animals were imported from breeding isolators into individually ventilated cages (IVCs) to minimize location effects. Oligo-MM12 animals to be colonized were administrated orally with approximately 10^9^ CFUs (in 200 µL). After 16 days, all animals were exported into a laminar flow hood and sacrificed. Cecal content was collected and snap frozen. Samples were kept at -80°C until they were ready for bile acid measurement and RNA extractions. RNA was extracted from 75-100 mg of cecal content obtained at the end of the experiment (16 days from the initiation of the colonisation experiment). 20 to 50 mg (dry weight) of cecal content were used for BA extraction. BA quantification and RT-qPCR assays were performed as described above.

### Animal housing and husbandry

Germ-free and gnotobiotic Oligo-MM12 [61] mice were generated by and housed in the Clean Mouse Facility (CMF, Department of Clinical Research of the University of Bern).

### Statistical analysis and data visualisation

Graphpad Prism 9.2.0 (GraphPad) was used to generate the figures shown in this paper and perform pairwise comparisons, the gene expression data was analysed with a linear model (LM) in R language v4.1.2 [62] using RStudio [63] a 2-way ANOVA or a Welch’s t-test. The statistical significance boundary was stablished at a *p*-value < 0.05.

### Supplementary Information

Supplementary Material 1. 

## Data Availability

The data used for this manuscript are publicly available in the following link: 10.5281/zenodo.6034320.
